# Diaminonaphthalene functionalized LUS-1 as a fluorescence probe for simultaneous detection of Hg^2+^ and Fe^3+^ in Vetiver grass and Spinach

**DOI:** 10.1038/s41598-024-66453-8

**Published:** 2024-07-16

**Authors:** Maryam Nouri, Leila Hajiaghababaei, Alireza Badiei, Faezeh Khalilian, Ali Mazloomifar

**Affiliations:** 1 Department of Chemistry, Yadegar-e-Imam Khomeini (RAH) Shahre Rey Branch, Islamic Azad University, Tehran, Iran; 2 School of Chemistry, College of Science, University of Tehran, Tehran, Iran

**Keywords:** 1,8-Diaminonaphthalene, Functionalized LUS-1, Fluorescence detection, Fe^3+^ and Hg^2+^, Vetiver grass, Spinach, Chemistry, Materials science

## Abstract

One of the important problems in the environment is heavy metal pollution, and fluorescence is one of the best methods for their detection due to its sensitivity, selectivity, and relatively rapid and easy operation. In this study, 1,8-diaminonaphthalene functionalized super-stable mesoporous silica (DAN-LUS-1) was synthesized and used as a fluorescence probe to identify Hg^2+^ and Fe^3+^ in food samples. The TGA and FT-IR spectra illustrated that 1,8-diaminonaphthalene was grafted into LUS-1. XRD patterns verified that the LUS-1 and functionalized mesoporous silica have a hexagonal symmetrical array of nano-channels. SEM images showed that the rod-like morphology of LUS-1 was preserved in DAN-LUS-1. Also, surface area and pore diameter decreased from 824 m^2^ g⁻^1^ and 3.61 nm for the pure LUS-1 to 748 m^2^ g⁻^1^ and 3.43 nm for the DAN-LUS-1, as determined by N₂ adsorption–desorption isotherms. This reduction demonstrated that 1,8-diaminonaphthalene immobilized into the pore of LUS-1. The DAN-LUS-1 fluorescence properties as a chemical sensor were studied with a 340/407 nm excitation/emission wavelength that was quenched by Hg^2+^ and Fe^3+^ ions. Hg^2+^ and Fe^3+^ were quantified using the fluorescence response in the working range 8.25–13.79 × 10^–6^ and 3.84–10.71 × 10^–6^ mol/L, with detection limits of 8.5 × 10^–8^ M and 1.3 × 10^–7^ M, respectively. Hg^2+^ and Fe^3+^ were measured in vetiver grass and spinach. Since the Fe^3+^ quenching can move in the opposite direction with sodium hexametaphosphate (SHMP) as a hiding compound for Fe^3+^, consequently, the circuit logic system was established with Fe^3+^, Hg^2+^, and SHMP as inputs and the fluorescent quench as the output.

## Introduction

Heavy metals in the environment, drinking water, and food are important problems for pollution control^1^. They are non-biodegradable metals, and their concentration in the environment is growing. Plentiful studies have emphasized metal toxicity in living organizations. Heavy metals are related to circumstances like neurotoxicity, bladder cancer, and genetic cell alteration^2^. Among heavy metals, Fe^3+^ has vital roles in biochemical procedures like numerous enzymic feedbacks, cell metabolism^3–5^. Fe^3+^ lack causes anemia^6^, feebleness^7^, diabetes^8,9^, and cancer^10,11^. Of course, excess Fe^3+^ damages DNA by creating free radicals and is the cause of Alzheimer's disease^12,13^ and Parkinson's disease^14^. Mercury is a hazardous metal, and low quantities of Hg^2+^ could increase the danger of ailments such as arrhythmia and kidney harm^15–18^. Therefore, there is a demand for novel systematic tools for simple, rapid, and selective monitoring of Fe^3+^ and Hg^2+^ ions^19–21^. Fluorescence methods and fluorescent sensors could accomplish these necessities^22^. Among the many detection procedures that have some inherent restrictions, like the usage of harmful solvents and complicated sample pretreatments, fluorescence is one of the best selections due to its sensitivity, selectivity, and relatively rapid and easy operation^23,24^.

A number of fluorescence sensors have already been made to detect Fe^3+^ or Hg^2+^^25–29^. For example, Ye et al.^25^ developed a new probe in terms of rhodamine B able to detect Hg^2+^ in living cells and colorimetrically detect Cu^2+^ in aqueous solution. A fluorescence probe was synthesized by Zhang et al.^26^ based on simple Schiff to detect Cu^2+^ and Hg^2+^ with a very low detection limit and analyze them in tap water and wastewater. In other studies, Iraqui et al. prepared starch-capped CdS quantum dots (QDs) for selective sensing of Hg^2+^ and Cu^2+^ in an aqueous medium^27^. Also, Uddin fabricated a paper-strip probe using CdS QDs stabilized with starch to detect Hg^2+^ and Cu^2+^^28^. The luminescence property of the CdS QDs is quenched after contact with Hg^2+^ and Cu^2+^. According to Wang et al.^29^ and Afshani et al.^30^, using the grafting of bis-Schiff base N,N′-(1,4-phenylenedimethyli-dyne)bis(1,4-benzenediamine) and salicylaldehyde with SBA-15, Fe^3+^ can be sensed at a less intense blue-emission peak. Also, Ghulam Fahmi et al.^31^ reported hybrid fluorescent chemosensors with emission in the green-to-red area utilizing N’-(5-nitro-2-oxoindolin-3-ylidene)thiophene-2-carbohydrazide modified SBA-15 to detect Fe^3+^. But only a few fluorescent probes exist for the simultaneous detection of Hg^2+^ and Fe^3+^^32–36^, and it seems useful to introduce better fluorescence probes that allow the simultaneous detection of Fe^3+^ and Hg^2+^.

As an ordered mesoporous silica, LUS-1 has a high surface area and hydrothermal constancy, which can be utilized in various fields^37–42^. It seems to be more attractive than the sensor scaffold. Though it itself is non-fluorescent, pores are covered with hydroxyl groups, acting as binding sites for the covalent grafting of organic compounds. If these organic compounds have good fluorescence properties, then a fluorescence sensor can be prepared in this way. On the other hand, naphthalenes with a donor-π-acceptor construction have fluorophore and chromophore properties^43^. Diaminonaphthalene has –NH_2_ group, which can form a complex with some ions^44^. It can be choosen as a fluorescent chemosensor because of its quick fluorescence lifetime and capability to be a donor and acceptor^45^.

In this regard, 1,8-diaminonaphthalene functionalized mesoporous silica (DAN-LUS-1) was synthesized and characterized. Then, DAN-LUS-1 was used as an original fluorescence chemosensor for simultaneous detection of Fe^3+^ and Hg^2+^. In addition, the circuit logic system was established with Hg^2+^, Fe^3+^, and sodium hexametaphosphate as inputs and the fluorescent quench as the output.

## Results and discussion

### Preparation and characterization mesoporous compounds

To synthesize the DAN-LUS-1, the LUS-1 was functionalized with chloropropyltrimethoxysilane to yeild LUS-Pr-Cl, reacting with 1,8-diaminonaphthalene to create DAN-LUS-1. The mesoporous compounds were characterized by SEM, XRD, FT-IR, BET and TGA.

**Figure 1:**
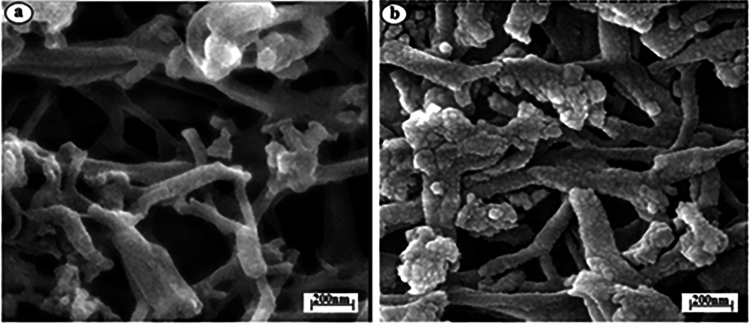
SEM micrograph of (**a**) LUS-1 and (**b**) DAN-LUS-1.

The morphology of LUS-1 and DAN-LUS-1 was investigated by SEM micrographs. Figure 1a shows SEM micrograph of the unmodified LUS-1 and Fig. 1b shows SEM micrograph of the DAN-LUS-1. SEM images showed that the LUS-1 has a rod-like morphology which is preserved in DAN-LUS-1^46^. Also, in Fig. 1b, it is clear that the 1,8-diaminonaphtalene is dispersed on the surface of channels.

**Figure 2:**
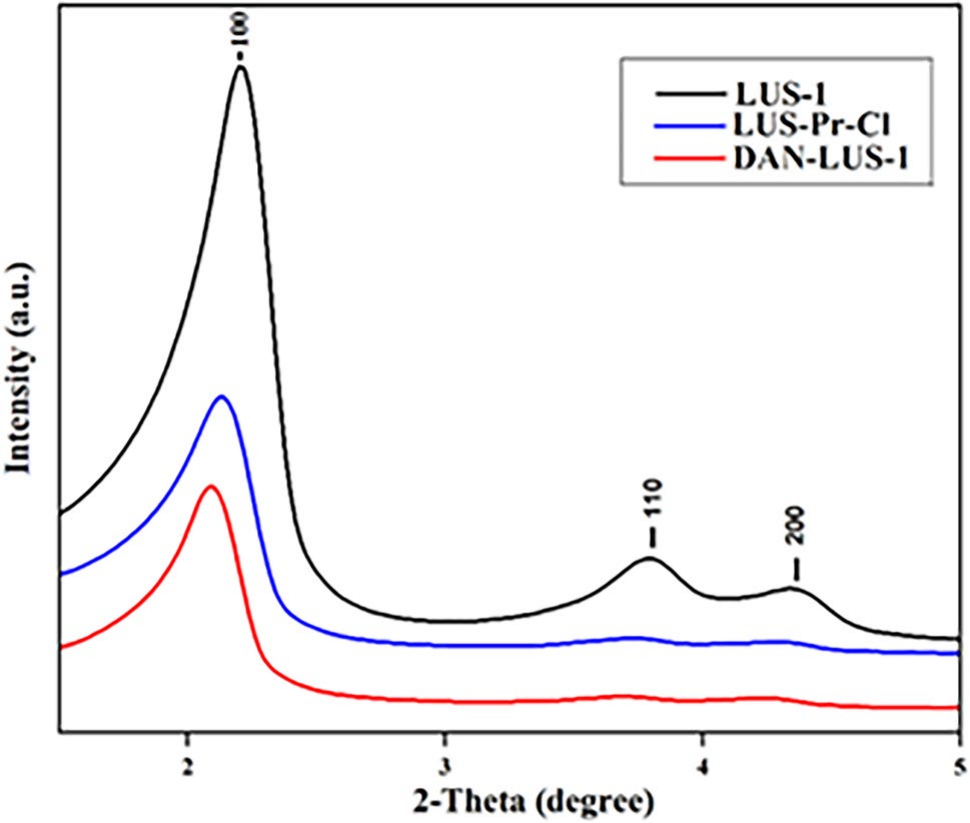
XRD spectrum of LUS-1, LUS-Pr-Cl, DAN-LUS-1.

XRD pattern of LUS-Pr-Cl, LUS-1, and DAN-LUS-1 displayed the same ordered, (200), (110), and (100) reflections, which displayed a hexagonal symmetrical array of nano-channels (Fig. 2). It is suggested that during the surface modification procedure, the LUS-1’s hexagonal structure is preserved, with no pore wall collapse. The peak strength (100) was reduced with a marginal peak shift after immobilizing the functionalized LUS-1 materials. The reason is the pore-wall scattering contrast difference and the organic groups irregular coverage on nanochannels^47,48^.

**Figure 3:**
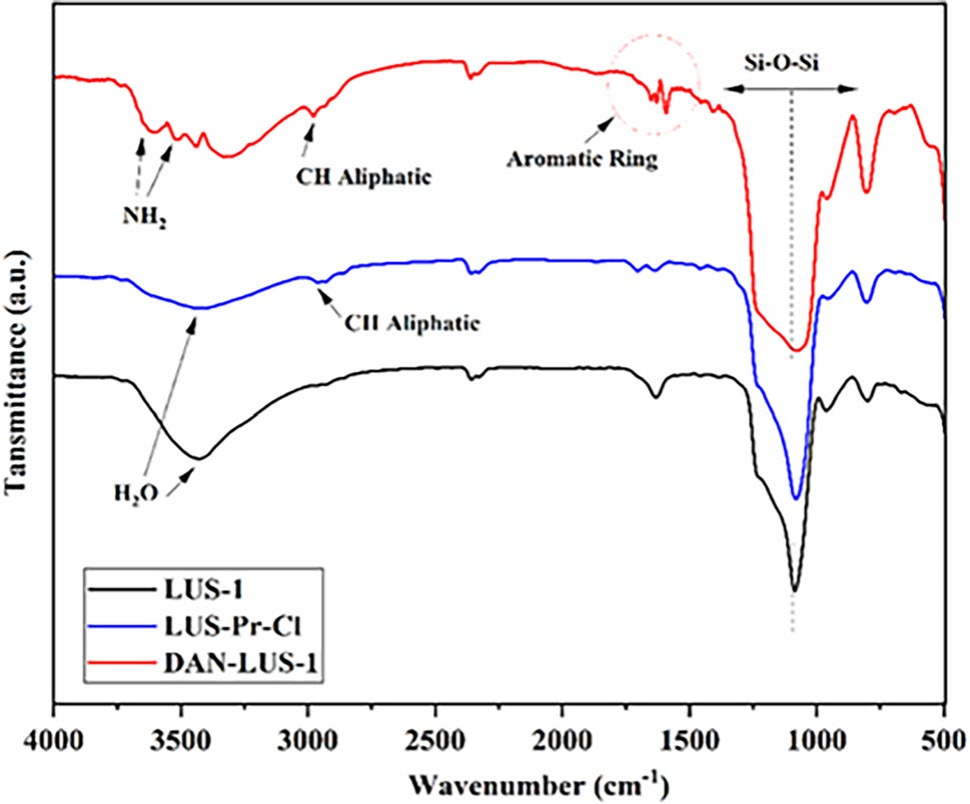
FT-IR spectrum of DAN-LUS-1, LUS-Pr-Cl and LUS-1.

Figure 3 gives the FT-IR spectra of LUS-Pr-Cl, LUS-1, and DAN-LUS-1. Mesoporous show peaks at 960, 800, 1100, and 3000–3450 cm^−1^ which belong to symmetric stretching of Si–O, asymmetric stretching vibrations of Si–O–Si, symmetric stretching of Si–OH, and stretching vibrations of OH, respectively^48^. Peak at 1650 cm^−1^ related to the physisorbed H_2_O onto the LUS-1. In LUS-Pr-Cl and DAN-LUS-1 spectra, peaks at 2880–2990 cm^−1^ are seen which are associated with the stretching vibrations of –CH_2_–of propyls^49^. Also, new peaks at around 3550 cm^−1^, 1580 cm^−1^ and 1700 cm^−1^ which are seen in DAN-LUS-1 spectrum, are assigned to the NH stretching vibration, NH bending^50,51^ and C=C aromatic stretching vibrations^52^. These new peaks confirm the successful attachment process.

**Figure 4:**
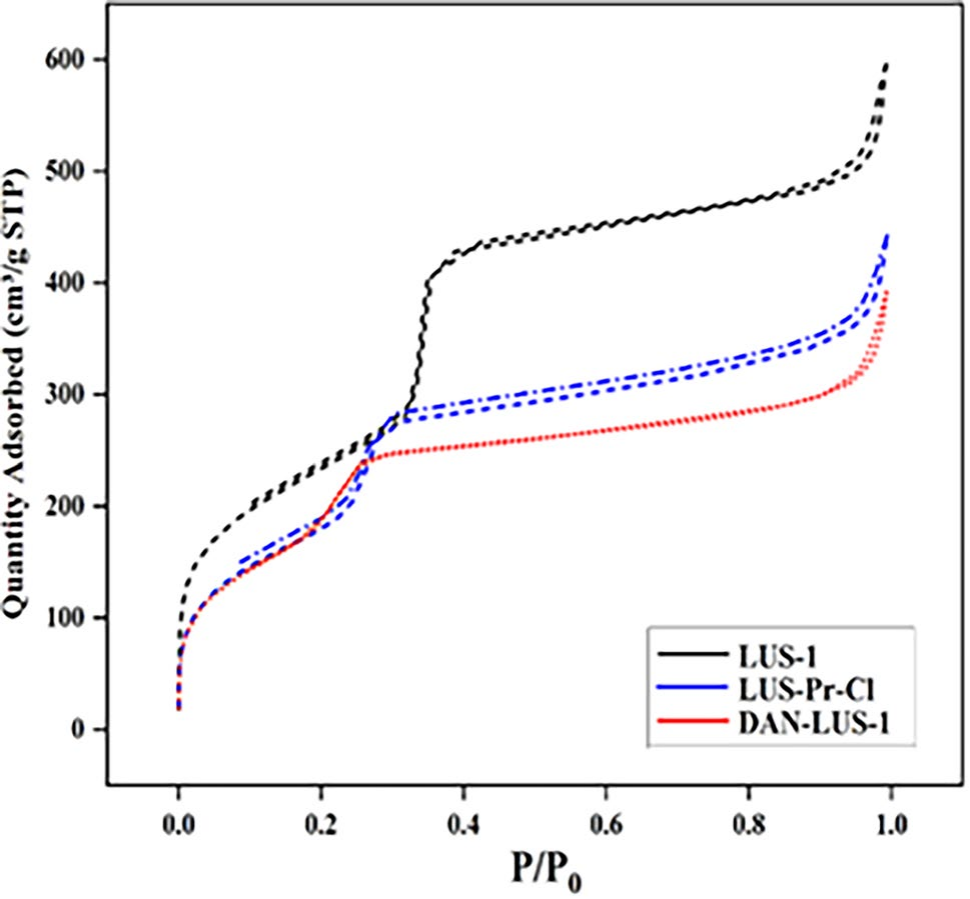
Nitrogen absorption–desorption diagram related to LUS-1, LUS-Pr-Cl, and DAN-LUS-1 compounds.

Figure 4 displays the N_2_ adsorption–desorption isotherms of LUS-Pr-Cl, LUS-1, and DAN-LUS-1 compounds. All the materials display “type IV” isotherm with an “H1-kind” hysteresis representative of the cylindrical construction which is related to mesoporous compounds matching IUPAC adsorption–desorption isotherms^53^.

Table 1 offers the samples' textural parameters counting pore diameter (BJH), specific surface area (BET), and total pore volume. The decrease in parameters indicates the effective attachment of organic parts on the inner surface.

**Table 1:** The porosimeter values for LUS-1 and DAN-LUS-1.

Modified surface	Surface area (m^2^/g)	Pore volume (cm^3^/g)	Pore diameter (nm)
LUS-1	824	0.79	3.61
DAN-LUS-1	748	0.49	3.43

**Figure 5:**
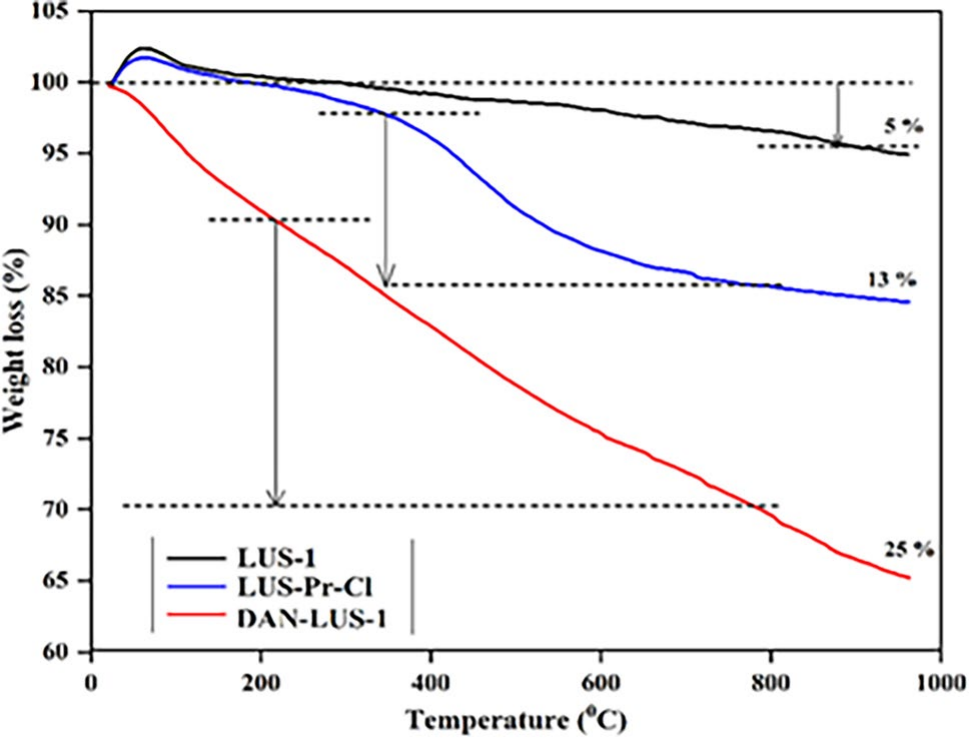
The TGA curves of LUS-Pr-Cl, LUS-1, and DAN-LUS-1.

The thermogravimetry analysis was done to evaluate the organic content in LUS-Pr-Cl, LUS-1, and DAN-LUS-1. Figure 5 offers TGA curves of all of them. The early increase in the LUS-1 and LUS-Pr-Cl weight from 100% was associated with the buoyancy effect^46^. The first weight loss was less than 150 °C owing to the evaporation of water on the surface^48,54^. Thus, the considerable weight losses between 200 and 800 °C associated with the decomposition of the organic species^52^. In this range, the weight loss for LUS-Pr-Cl, and DAN-LUS-1 was related to the attached chloropropyl trimethoxysilane (13%), and the attached 1,8-diaminonaphthalene (25%), respectively.

### Fluorescence studies

**Figure 6:**
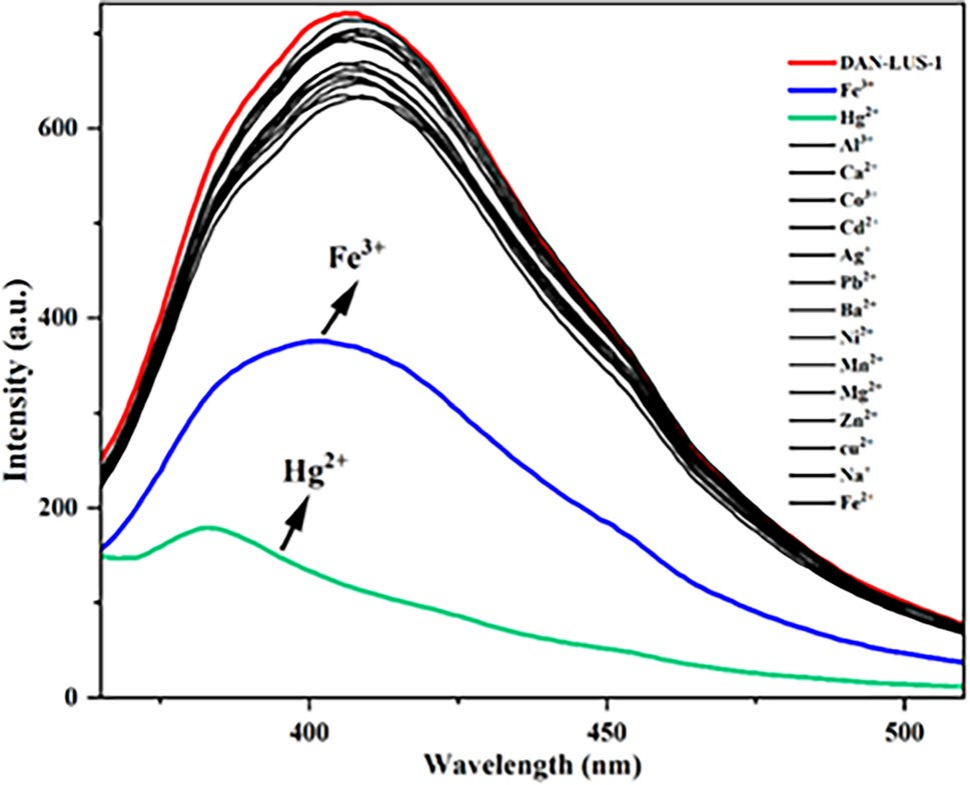
Fluorescence emission spectra of the suspension of DAN-LUS-1 in water (0.05 g L^−1^) after adding Hg^2+^, Fe^3+^, Al^3+^, Ca^2+^, Co^3+^, cd^2+^, Ag^+^, Pb^2+^, Ba^2+^, Ni^+^, Mn^2+^, Mg^2+^, Zn^2+^, Cu^2+^, Na^+^ and Fe^2+^ ions (λ_ex =_ 340 nm, λ_em_ = 407 nm).

DAN-LUS-1 was applied as a fluorescence sensor to determination of Hg^2+^ and Fe^3+^ ions in water samples. The emission spectra of a dilute suspension of DAN-LUS-1 in water (0.05 g L^−1^) were investigated at the excitation wavelength (λex) 340 nm. There was an emission peak at 407 nm (Fig. 6). Adding various metal ions to DAN-LUS-1 suspension changed the emission intensity. A significant reduction in the amount of the emission signal at 407 nm was observed only after addition of Hg^2+^ and Fe^3+^ ions and then changed partially by adding Al^3+^, Ca^2+^, Co^2+^, Cd^2+^, Ag^+^, Pb^2+^, Ba^2+^, Ni^2+^, Mn^2+^, Mg^2+^, Zn^2+^, Cu^2+^, Na^+^ and Fe^2+^ ions (Fig. 6). These findings indicate that DAN-LUS-1 can be used as a fluorescence sensor for detecting and determining Hg^2+^ and Fe^3+^ ions in aqueous media. Quenching effect of metal ions can be attributed to the paramagnetic nature of these ions or the heavy metal ion effect^55^.

**Figure 7:**
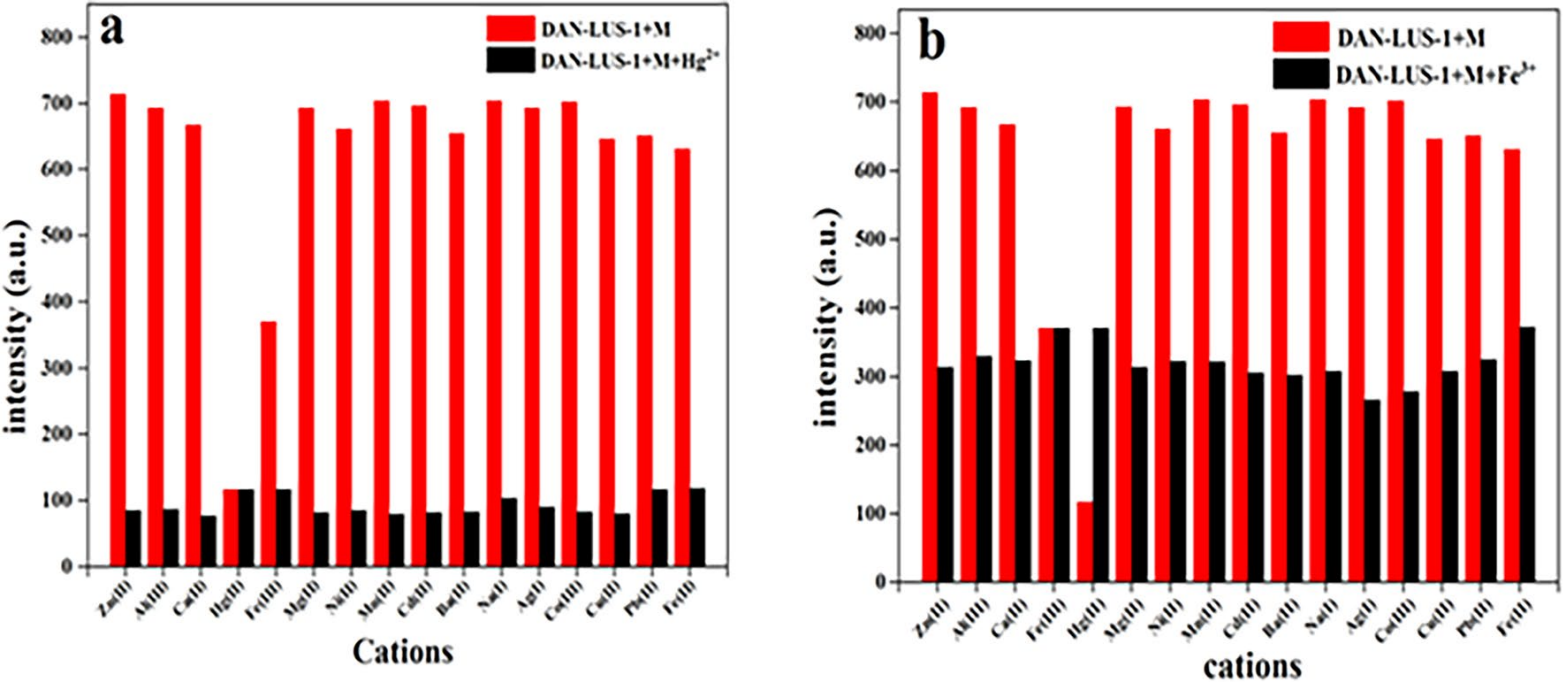
Competition test of DAN-LUS-1 (2mL water suspension, 0.05 g L^−1^) for (**a**) Hg^2+^ (50 μL, 1 × 10^–2^ M) and (**b**) Fe^3+^ (50 μL, 1 × 10^–2^ M) in the existence of other cations (50 µL of 1 × 10^–2^ M) (λex = 340 nm, λem = 407 nm).

The selectivity toward Hg^2+^ and Fe^3+^ ions was examined when competitive metal ions such as Al^3+^, Ca^2+^, Co^2+^, Cd^2+^, Ag^+^, Pb^2+^, Ba^2+^, Ni^2+^, Mn^2+^, Mg^2+^, Zn^2+^, Cu^2+^, Na^+^ and Fe^2+^ exist in aqueous DAN-LUS-1 suspension (0.05 g/L) (Fig. 7). The emission spectra were recorded followed by a mixture of 50 µL Hg^2+^ or Fe^3+^ (0.01 M) and 50 µL M^n+^ (0.01 M) solution was applied to 2 mL of DAN-LUS-1 suspension. The reported results in Fig. 7a,b show that DAN-LUS-1 is a selective chemosensor for Hg^2+^ and Fe^3+^ ions, amongst the other cations.

**Figure 8:**
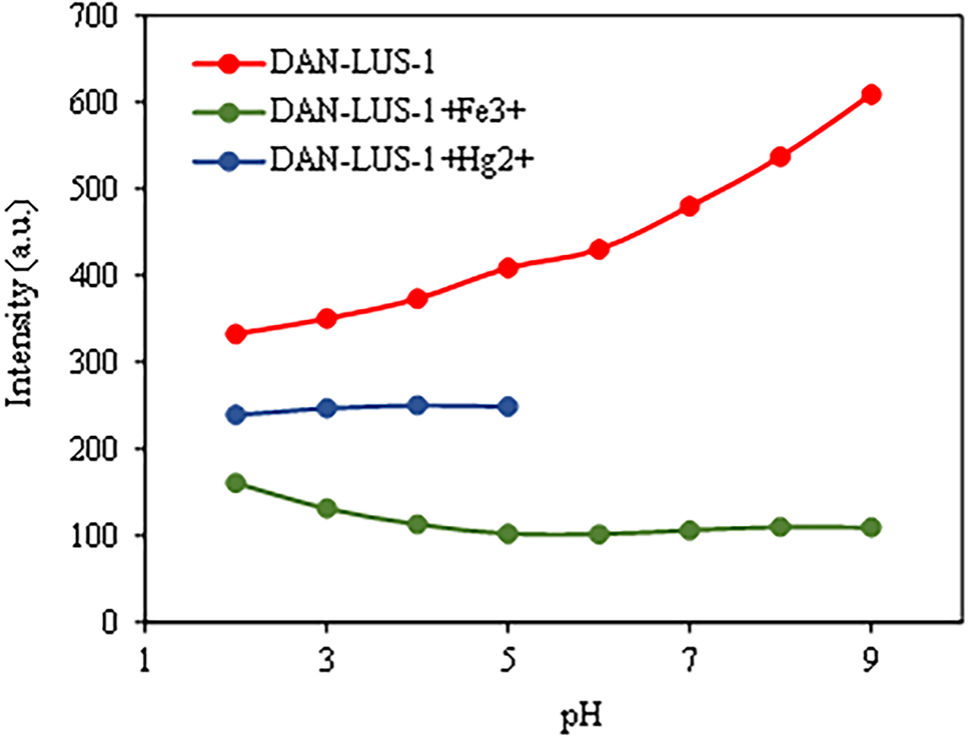
Effect of the pH on the fluorescence intensity of (**a**) DAN-LUS-1, (**b**) DAN-LUS-1 + Fe^3+^ and (**c**) DAN-LUS-1 + Hg^2+^.

The fluorescence behavior of the suspension of DAN-LUS-1 in absence and presence of Hg^2+^ over the pH range 2–9 and in presence of Fe^3+^ over the pH range 2–5 was studied. The pH of the solutions was adjusted by adding HCl or NaOH. As can be seen in Fig. 8, with addition of Fe^3+^ and Hg^2+^, a significant fluorescence quenching was occurred. Fluorescence intensity of DAN-LUS-1 (at 407 nm) increases with increasing pH. The quenching of Hg^2+^ increases with the increase of pH from 2 to 5 and remains almost constant after that. The quenching Fe^3+^ is almost constant in the range of pH 2 to 5. Therefore, in the simultaneous measurement of Hg^2+^ and Fe^3+^, pH = 5 is the best pH. Of course, if the goal is only to measure Hg^2+^, a higher pH where the fluorescence intensity of the DAN-LUS-1 is higher can be used to increase the sensitivity of the measurement.

**Figure 9:**
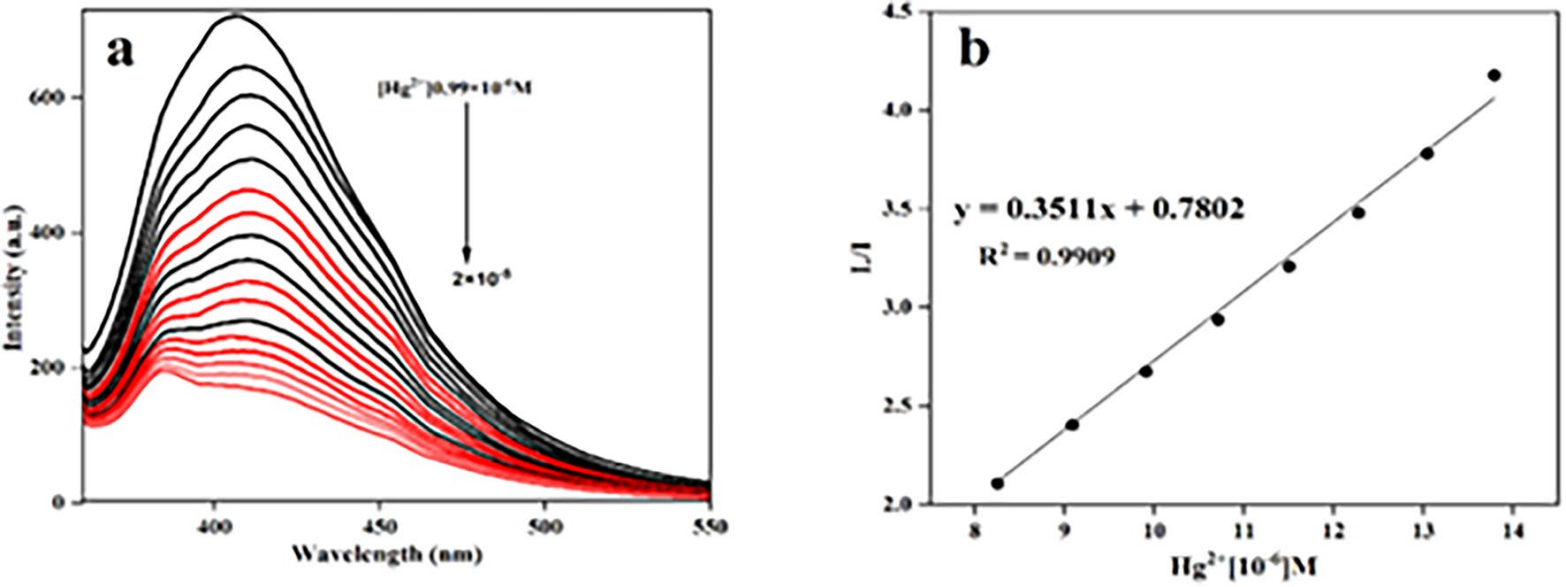
(**a**) Fluorescence titration of DAN-LUS-1 in the presence of various concentrations of Hg^2+^ ions. (**b**) calibration curve to detect Hg^2+^ (λex = 340 nm, λem = 407 nm).

**Figure 10:**
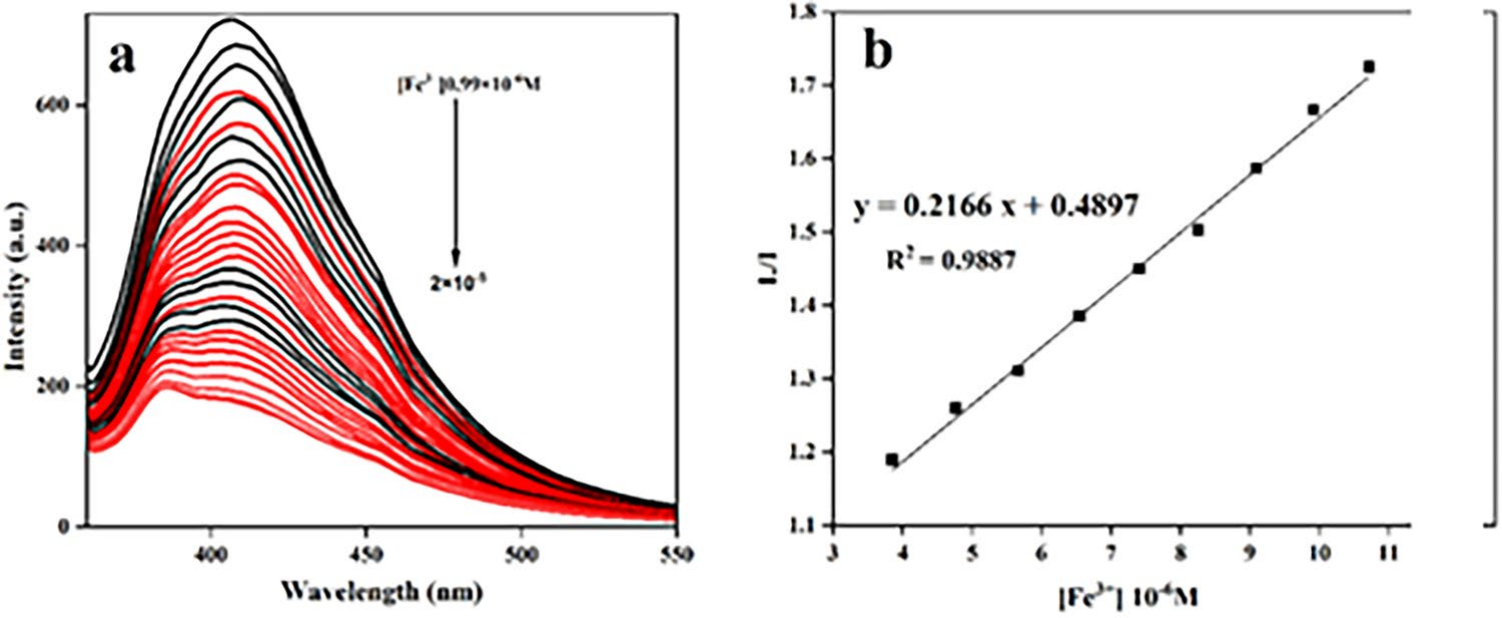
(**a**) Fluorescence titration of DAN-LUS-1 in the presence of various concentrations of Fe^3+^ ions. (**b**) calibration curve to detect Fe^3+^ (λex = 340 nm, λem = 407 nm).

The recognition capacity of DAN-LUS-1 toward Hg^2+^ and Fe^3+^ ions was determined in the aqueous solution of the DAN-LUS-1 suspension via titration test. When the Hg^2+^ and Fe^3+^ ions concentration increases (Figs. 9a and 10a), the fluorescence intensity reduces. The Stern–Volmer plots were revealed in Figs. 9b and 10b for Hg^2+^ and Fe^3+^, respectively. A linear relation between fluorescence intensity and Hg^2+^ and Fe^3+^ ions concentration was obtained by the equations y = 0.3511x + 0.7802 and y = 0.2166x + 0.4897 with a regression coefficients, R^2^ = 0.9909 and R^2^ = 0.9887, respectinely. The linear response spans a concentration of 8.25–13.79 × 10^–6^, 3.84–10.71 × 10^–6^ mol/L of Hg^2+^ and Fe^3+^, respectinely. Moreover, the detection limit was based on the DL = 3S_d_/m equation, in which S_d_ shows the standard deviation of blank and m represents the slope of calibration curve (Stern–Volmer plot). The detection limits were 8.5 × 10^–8^ M and 1.3 × 10^–7^ M for Hg^2+^ and Fe^3+^ determination, respectively.

Recyclability is a highly important criterion for assessing the fluorescence sensor's performance in practical applications. The utilized DAN-LUS-1 are easily extracted from the solution using a fast centrifugalizing, repeatedly cleaned in distilled water, and then dried at 60 °C. Recycled DAN-LUS-1 was re-dispersed in the reaction media for subsequent experiments. After three consecutive cycles, the fluorescence of reused DAN-LUS-1 showed no detectable change. This indicates the stability and reusability of the proposed DAN-LUS-1 sensor as a fluorescence sensor towards Hg^2+^ and Fe^3+^.

### Mechanism of quenching

**Figure 11:**
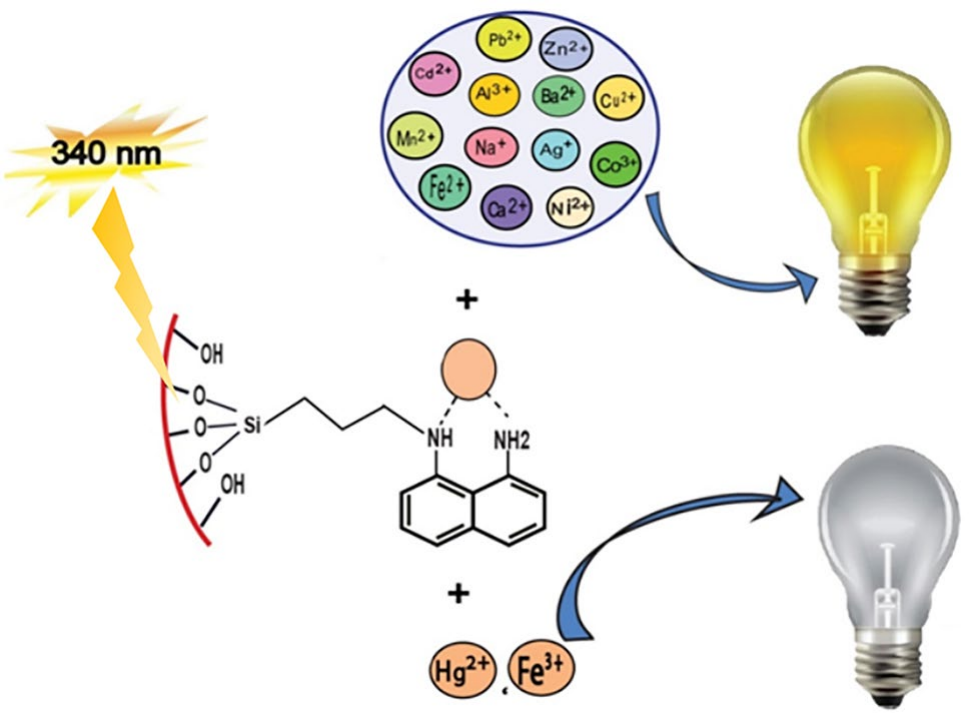
Schematic of the DAN-LUS-1 quenching mechanism in the presence of Hg^2+^ and Fe^3+^.

The higher sensitivity of DAN-LUS-1 toward Fe^3+^ and Hg^2+^ ions is possibly caused by their fast chelating kinetics and higher affinity with nitrogen atoms of the probe. Unmodified LUS-1 is non-fluorescent, but 1,8-diaminonaphthalene is a fluorescent molecule. When LUS-1 is functionalized with 1,8-diaminonaphthalene, DAN-LUS-1 will also be fluorescent. Therefore, the emission bands are from 1,8-diaminonaphthalene molecules. The quenching of the fluorescence emission can be attributed to the complex formation between metal ions and DAN-LUS-1 as a ligand molecules. DAN-LUS-1 most likely binds with Hg^2+^ and Fe^3+^ via the nitrogen atoms of functional group of 1,8-diaminonaphthalene in mesoporous framework which potentially leading to quenching effect due to the photoinduced electron transfer (PET) mechanism^56^. Electron transfer occurs from the excited DAN-LUS-1 (as a Lewis base) to the electron-deficient metal ions (as a Lewis acid).Also, the observed quench created by Fe^3+^ is possible their paramagnetic nature. Forbidden intersystem crossings are simplified by paramagnetic species, leading to quenching the fluorescence emission. The heavy atom effect can be considered to explain fluorescence quenching by Hg^2+^^57^. Figure 11 indicates the proposed fluorescence sensing mechanism of DAN-LUS-1 by Hg^2+^ and Fe^3+^.

### Logic behaviour of DAN-LUS-1

**Figure12:**
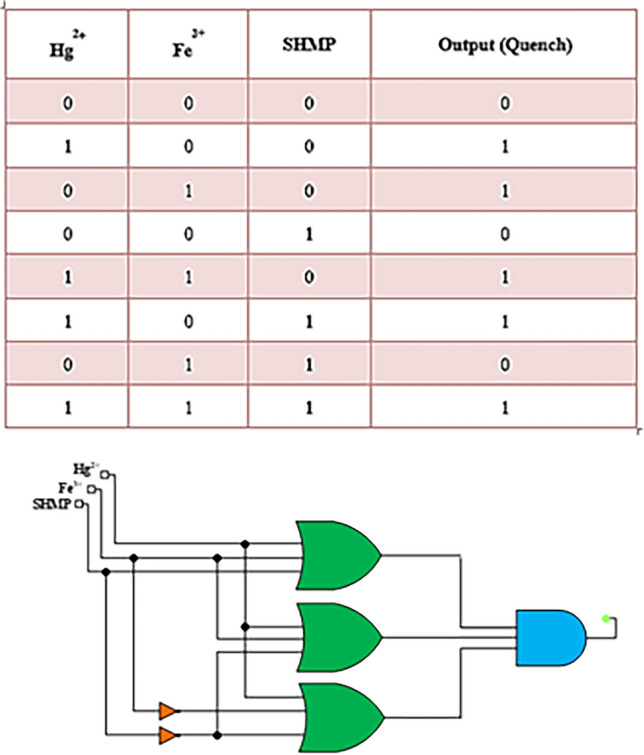
The schematic of the truth table and logic circuit for DAN-LUS-1 with Hg^2+^, Fe^3+^, and SHMP as the inputs.

A binary logic circuit can be made from the correspondence between Hg^2+^, Fe^3+^, and sodium hexametaphosphate (SHMP) as the inputs and the fluorescent quench of DAN-LUS-1 at 407 nm as the output (Fig. 12). SHMP is a masking agent to block Fe^3+^. The circuit was deliberate according to the standard expression of products-of-sum. The Products-of-sum generate the truth table, where the presence and absence of the inputs were illustrated by 0 and 1, respectively. Concerning the output, 0 displays a fluorescence emission, while 1 clarifying an emission quenching. Consequently, five out of eight probable combinations led to a fluorescence quenching.

**Figure 13:**
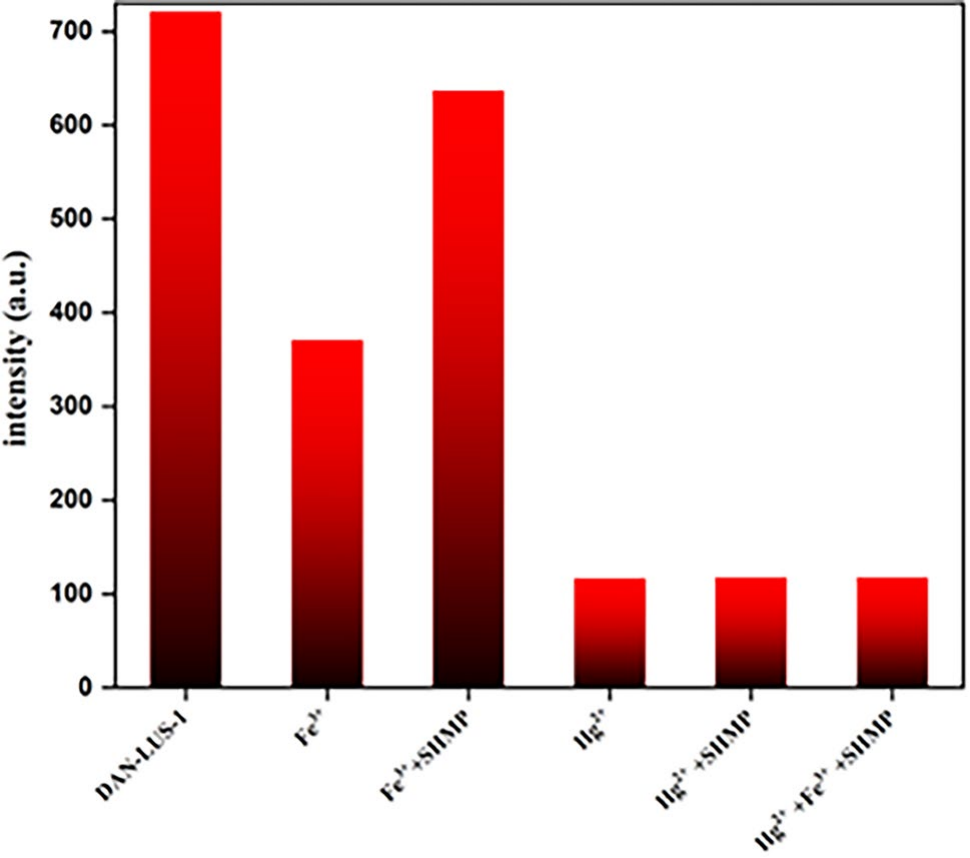
Hiding effect of sodium hexametaphosphate (SHMS) for Fe^3+^.

Hiding effect of SHMS for Fe^3+^ was investigated with adding Fe^3+^ and SHMS to DAN-LUS-1. The probe can be switched from "on" to "off" by adding SHMP to a solution containing DAN-LUS-1 and Fe ^3+^. As can be seen in Fig. 13, the Fe^3+^ quenching can move in the opposite direction with adding SHMP as a hiding compound. But, the Hg^2+^ quenching does not change with adding SHMP. These results enable the simultaneous detection of Fe^3+^ and Hg^2+^ using a chemosensor and a logic circuit.

### Determination Hg^2+^ and Fe^3+^ in Vetiver Grass and Spinach

The proposed fluorescence sensor and Inductively Coupled Plasma-Mass Spectrometry (ICP-MS) were applied to determine Fe^3+^ and Hg^2+^ in vetiver grass and spinach. The results obtained are presented in Table 2. Good agreement between the two methods indicate the capability of the proposed methode for the determination of Fe^3+^ and Hg^2+^ in the food samples and approve the accurateness of the suggested technique and its independency from matrix effects.

**Table 2:** The detected concentrations of Hg^2+^ and Fe^3+^ in real specimens by proposed sensor and ICP-MS.

Real samples	Detected ion	Detected concentration by ICP-MS	Detected concentration by proposed sensor
**Spinach**	Fe^3+^	1.2 (µg/g)	1.1 (µg/g)
**Vetiver grass**	Hg^2+^	10.0 (µg/g)	9.5 (µg/g)

### Comparison section

A comparison was made between the several Fe^3+^ and Hg^2+^ fluorescence sensors using various sensing materials and the proposed sensor (DAN-LUS-1) developed in this study (Table 3)^58–62^. Overall, the developed fluorescence sensor is superior in detection limit to the mentioned sensors. Also, the proposed sensor is one of the few sensors that allows the simultaneous detectionof Fe^3+^ and Hg^2+^.

**Table 3:** Comparison with the other Fe^3+^ and Hg^2+^ fluorescence sensors.

Fluorescence probe	Structure	Fe^3+^	Hg^2+^	Detection limit (mol L^−1^)	Logic gate	Ref. no
SBA-functionalized by calixarene		** × **	✓	3.3 × 10^–7^	** × **	^58^
MCM-3T		** × **	✓	8.0 × 10^–6^	** × **	^59^
SBA-15/ APTES-NH		✓	** × **	1.49 × 10^–3^	** × **	^60^
PyU-SBA-15		** × **	✓	9.92 × 10^–8^	** × **	^61^
g-C_3_N_4_		✓	✓	Fe^3+^ = 1.9 × 10^–7^ Hg^2+^ = 1.2 × 10^–8^	** × **	^62^
LUS-Pr-DAN		✓	✓	Fe^3+^ = 1.3 × 10^–7^ Hg^2+^ = 8.5 × 10^–8^	✓	This work

## Experimental part

### Reagents and apparatus

All materials and tools are describes in Sect. S1.

### Synthesis of DAN-LUS-1

**Figure 14:**
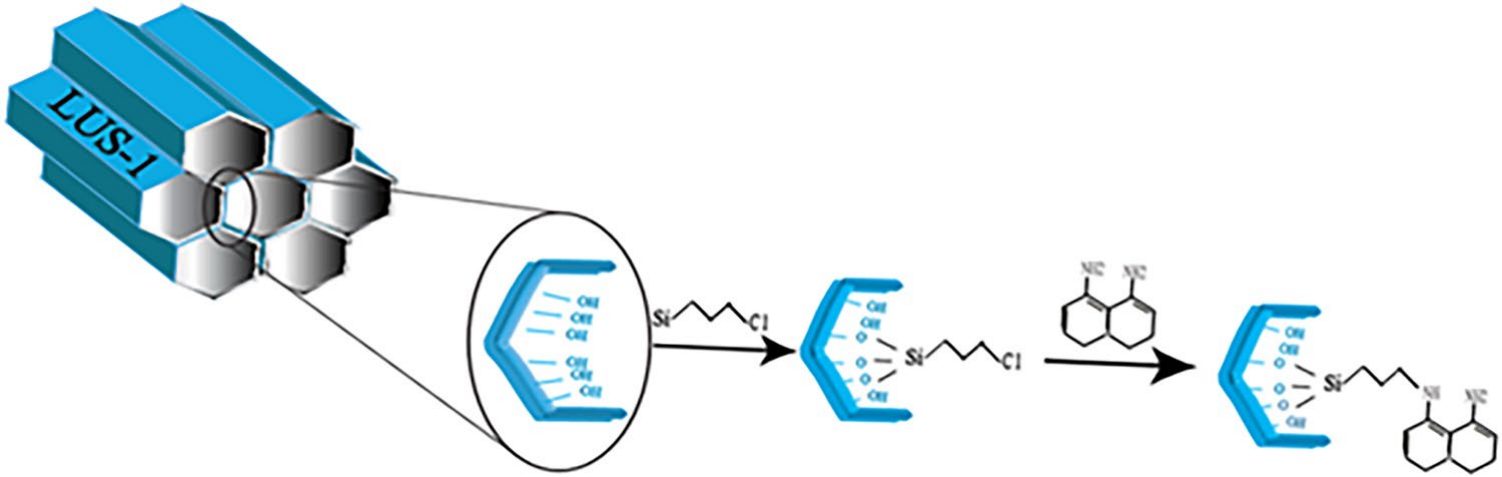
The synthesis procedure of DAN-LUS-1.

DAN-LUS-1 was synthesized in three steps. At first, LUS-1 was synthesised in terms of the process previously reported by Rahimifard et al*.*^37^ It describe in Sect. S1. After that, 3-Chloropropyltrimethoxysilane (7 mL) was added to LUS-1 (7 g) in 150 mL dried toluene and refluxed for 24 h and soxhlet in EtOH to give LUS-Pr-Cl compound. Finaly, triethylamine (5 mL), LUS-Pr-Cl (1g), and 1,8-diaminonaphthalene (0.1 g) were refluxed in toluene (40ml) for 24 h and soxhlet in EtOH to give DAN-LUS-1 (Fig. 14).

### Analytical procedure

Fluorescence measurements for ions (Hg^2+^, Fe^3+^, Al^3+^, Ca^2+^, Co^2+^, Cd^2+^, Ag^+^, Pb^2+^, Ba^2+^, Ni^2+^, Mn^2+^, Mg^2+^, Zn^2+^, Cu^2+^, Na^+^ and Fe^2+^) were performed at room temperature. 5 mg of DAN-LUS-1 as an optical probe was distributed in deionized water (100 mL). Then, the fluorescence quartz cell was filled with suspension solution (2 mL) and different amounts of the solution of ion. The fluorescence data of the samples were attained within the wavelength range of 250–600 nm and excitation wavelength of 340 nm.

### Hg^2+^ and Fe^3+^ determination in real samples

Vetiver grass and spinach were utilized as real samples to use the fluorescence probe and determine Hg^2+^ and Fe^3+^. In this process, for 3 months period, all vetiver grass was watered with Hg^2+^ (30 mg/L) solution. After this period, they were cutted and washed. Then they were dried at 70 ℃ in an oven. Dried vetiver (5g) was digested with 15 mL hydrochloric acid (HCl) and 5 mL nitric acid (HNO_3_), and 10 mL hydrogen peroxide (H_2_O_2_). The sample was heated on hot plate within the fume chamber during the digestion procedure, for 2 h and each hour 10 mL of the solution were added owing to drying the sample. H_2_O_2_ was added little by little during the digestion process. After finishing the digestion process, samples were cooled down and pH adjusted by NaOH. The ultimate volume of the solution was set to 25 mL with distilled water. Spinach was dried and digested according to vetiver digestion method. All procedures and experiments related to the vetiver grass and spinach are complied with relevant institutional, national, and international guidelines and legislation.

## Conclusion

In this work, a novel fluorescent sensor was prepared in terms of the surface modification of LUS-1 by a 1,8-diaminonaphthalene (DAN-LUS-1) for high sensitive detection of Hg^2+^ and Fe^3+^ ions. The DAN-LUS-1 was synthesized and characterized. FT-IR and TGA spectra represented the presence of 1,8-diaminonaphthalene groups in the silica system. The XRD pattern displayed a hexagonal symmetrical array of nano-channels suggested that DAN-LUS-1 was characteristic of mesoporous silica. The SEM images of mesoporous materials represented the rope-shaped morphology. Consequently, Hg^2+^ and Fe^3+^ were quantified using the fluorescence response at 407 nm in working range 8.25–13.79 × 10^–6^ and 3.84–10.71 × 10^–6^ mol/L, respectively. Detection limits were 8.5 × 10^–8^ and 1.3 × 10^–7^ mol L^−1^ for Hg^2+^ and Fe^3+^ determination, respectively. The introduced sensor was successfully applied to measure the concentration of Hg^2+^ and Fe^3+^ ions in the Vetiver Grass and Spinach. Satisfactory results were obtained, which were consistent well with the results from the standard ICP-MS technique.

Under the individual actions of the of Hg^2+^ and Fe^3+^, and their combination, a solid support molecular logic circuit was obtained successfully, producing one optical outputs while stimulating three chemical inputs. In this arrangement, a combinatorial system is developed simultaneously acting as a logic circuit and as a chemosensor in terms of grafted molecules on the solid support.

The results revealed a fast and easy method to determine Hg^2+^ and Fe^3+^ at trace levels and construct a combinatorial logic circuit.

### Supplementary Information

Supplementary Information.

## Data Availability

The datasets generated during and/or analysed during the current study are available from the corresponding author on reasonable request.
