# Supplementary material for: Diaminonaphthalene functionalized LUS-1 as a fluorescence probe for simultaneous detection of Hg2+ and Fe3+ in Vetiver grass and Spinach
Source: Sci Rep. 2024 Jul 16;14:16376. doi: 10.1038/s41598-024-66453-8 (PMC11252323; doi:10.1038/s41598-024-66453-8)
Supplement: Supplementary file 1 — Supplementary Information. [file 41598_2024_66453_MOESM1_ESM.docx]

**S1**

**Diaminonaphthalene functionalized LUS-1 as a fluorescence probe for simultaneous detection of Hg^2+^ and Fe^3+^ in Vetiver grass and Spinach**

**Maryam Nouri^1^, Leila Hajiaghababaei^1,^*, Alireza Badiei^2,^*, Faezeh Khalilian^1^, Ali Mazloomifar^1^**

^1^Department of Chemistry, Yadegar-e-Imam Khomeini (RAH) Shahre Rey Branch, Islamic Azad University, Tehran, Iran

^2^School of Chemistry, College of Science, University of Tehran, Tehran, Iran

* Corresponding authors, e-mail: Leila Hajiaghababaei: [lhajiaghababaei@yahoo.com](mailto:lhajiaghababaei@yahoo.com),

[lhajiaghababaei@iausr.ac.ir](mailto:lhajiaghababaei@iausr.ac.ir)

Alireza Badiei: [abadiei@khayam.ut.ac.ir](mailto:abadiei@khayam.ut.ac.ir)

**Materials and Instruments.** The chemical materials which were employed in this work were obtained from Merck Company and were used with no purification. The solvents were spectrometric grade and were used as received. Stock solutions of all cations were prepared using their nitrate salts. Melting points were evaluated by the capillary tube method with an Electrothermal 9200 apparatus. Infrared (IR) spectra were obtained in KBr disks on a Fourier transform (FT-IR) Bruker Tensor 27 spectrometer 400-4000 cm^-1^ region. The thermogravimetric analyses (TGA) were collected on TGA Q50 V6.3 Build 189 instrument from rt to 1000 °C. FESEM analysis was performed using SIGMA VP instrument. Lowangle X-ray patterns were obtained Rigako Ultima IV instrument. N_2_ sorption analyses were conducted on Micromeritics tri star II Plus at -196 °C. Surface areas, pore volumes and sizes were collected using Brunaue-Emmet-Teller and Barrett Joyner Halenda methods by BELSORP software. Agilent G9800A’s Fluorescence Spectrometer used for fluorescence studies. ICP-MS measurements were performed using a Perkin Elmer Nexion 300X.

Silica gel 60, Sodium hydroxide, hexadecyltrimethylammonium-p-toluene-sulfonate, triethylamine and metal salts, (3-chloropropyl) Trimethoxysilane, 1,8-Diaminonaphthalene were supplied by Merck, ethanol, p-toluenesulfonic acid monohydrate and salts were purchased from Sigma Aldrich and used without any further purification.

**The synthesis of LUS-1.** LUS-1 was synthesized following a typical process previously reported by Rahimifard et al. 15.5 g (0.26 mol) of colloidal silica Ludox was added to a solution of 2 g (5 × 10^−2^ mol) sodium hydroxide in 50 mL distilled water while stirring until obtaining a clear solution, on the other side, by the dissolution of 2.5 g (5.5 × 10^−3^ mol) cetyltrimethylammonium toluene sulfonate in 90 mL of distilled water at 40 °C preparing another solution. By adding the former solution gradually (dropwise) to the second solution and stirring the mixture for 2 h at 40 °C, a sol-gel was obtained. Then, the gained sol-gel heated using autoclave for 24 h at 130 °C. Next, the surfactant was removed from the structure upon treatment with 0.1 M HCL in ethanol for 2 h. Finally, the obtained white powder was washed with distilled water and dried under vacuum at 100 °C.
